# Research on the Evolution of Shield Segment Cracks Based on Acoustic Emission and CMOD

**DOI:** 10.3390/ma15175829

**Published:** 2022-08-24

**Authors:** Junwei Li, Fei Xu, Tianmu Wang, Songtao Shi

**Affiliations:** 1 School of Civil Engineering, Shijiazhuang Tiedao University, Shijiazhuang 050043, China; 2 Key Laboratory of Large Structure Health Monitoring and Control, Shijiazhuang Tiedao University, Shijiazhuang 050043, China; 3 School of Civil Engineering, Southwest Jiaotong University, Chengdu 610031, China

**Keywords:** segment crack damage, acoustic emission, CMOD, crack evolution characteristics, segment cracking damage model

## Abstract

In order to explore the cracking law and failure characteristics of segments, a model test of shield segment cracking was conducted. The microscopic and macroscopic crack evolution process of the segment is studied by using acoustic emission detection technology and crack opening displacement (CMOD). According to the acoustic emission signal and CMOD, characteristics generated in the process of segment cracking, in the form of numerical value, the evolution characteristics of each stage of segment cracking are directly reflected. Based on acoustic emission energy and CMOD, the segment cracking damage model was established to determine the segment fracture damage degree. The result shows that segment cracking can be divided into three stages, and the acoustic emission detection results and CMOD have different degrees of change in each cracking stage. This proves that both the acoustic emission acquisition results and CMOD can be used as evaluation indicators of damage degree. Acoustic emission can accurately identify the crack evolution process, and the yield strengthening is an important stage of crack damage evolution. The damage data points in this stage account for 76.83% of all the damage data points, the occurrence rate of damage data points is 0.225 s, and the density of data points in the damaged area is 3.219 × 10^−4^ mm^3^, which is larger than the other two stages. The segment cracking damage model can effectively reflect the segment cracking degree and provide a reference for the actual segment cracking assessment.

## 1. Introduction

Since the 20th century, with the development of China’s infrastructure construction, the shield method [1] has been widely used in China’s tunnel construction due to its significant advantages such as high drilling efficiency, less personnel input, and less stratum disturbance. The main material of the segment is concrete, a typical heterogeneous composite material. When a segment crack occurs, it first gathers inside the concrete. As the load is applied, the crack spreads around and finally forms a through crack [2,3,4]. The occurrence of cracks will deteriorate the physical properties and durability of the segment structure to varying degrees and will lead to tunnel collapse in severe cases. Therefore, it is very important to conduct research on the evolution characteristics of segment structure cracking and to clarify the degree of segment structure deterioration.

At this stage, the research on the causes and mechanism of cracking of concrete materials is more focused on the surface cracks of concrete, and the evolution characteristics of cracking of concrete materials are reflected through the geometric characteristics of crack width, depth, and so on. Mandelbrot et al. [5,6,7] found that the shape of the fracture surface of concrete has fractal characteristics, and the fractal theory has been widely used in the study of concrete structures since then. Qin et al. [8,9] found that there is a good linear relationship between the development characteristics of concrete surface crack opening displacement (CMOD) and the physical and mechanical properties of concrete materials.

Xu et al. [10] conducted a wedge-type compact tensile concrete fracture test, and the calculation formula of CMOD is preliminarily derived. It proved that the CMOD corresponding to the crack initiation point and the instability point of concrete fracture increases linearly with the height of the specimen. Yin et al. [11,12] studied the influence of CMOD on the fracture performance of three-point bending concrete beams. It is concluded that the larger the initial crack height ratio is, the more the CMOD is affected by the initial crack height ratio. Yu et al. [13] compared the two methods for calculating the fracture energy of concrete with the load-crack opening displacement curve (P-CMOD) and the load-deflection curve (P-δ). It proved that the fracture energy of concrete calculated by the P-CMOD curve has less discreteness and better stability. As a commonly used eigenvalue, CMOD has been widely used in the study of concrete cracks, but CMOD is the surface eigenvalue of cracks, which cannot effectively reflect the evolution and development of internal cracks in concrete. There are also relatively few studies that perform quantitative analysis.

Acoustic emission technology, as a typical non-destructive testing technology, can display the elastic wave generated when the concrete structure is damaged in the form of an electrical signal. It then describes the whole process from micro-damage to macro-damage of concrete structure [14]. Li et al. [15] explored the influence of concrete materials with different aggregate particle sizes on the accuracy of acoustic emission detection through the acoustic emission lead breaking test of concrete materials. The optimal acoustic emission sensor arrangement and acoustic emission acquisition parameters are obtained. Guo et al. [16,17], according to the load-displacement curve analysis of acoustic emission ringing count rate and energy rate, found that the acoustic emission signals of C30 and C40 concrete (medium strength concrete) are in the whole process of compression, and the acoustic emission activity period is at the ultimate load. In recent years, more scholars have begun to use the variation of acoustic emission energy and cumulative event number of acoustic emission to describe the failure characteristics of segments under force and the damage characteristics at various stages under different assembly methods, capping block positions, and the number of cracks [18,19,20,21]. However, a large number of scholars only use the acoustic emission energy and the cumulative event number of acoustic emissions to study segment damage, while ignoring the advantages of acoustic emission localization to describe the distribution of micro-damage and damage range.

In view of this, a segment failure model test was performed based on a shield tunnel in a metro area. Through the acquisition of the acoustic emission signal and CMOD of the whole process of the segment from microscopic to macroscopic, the evolution law of segment cracking is explored. The segment cracking damage model is established by combining the acoustic emission signal and CMOD with the damage mechanics theory.

## 2. Acoustic Emission Technology and Basic Theory of CMOD

### 2.1. Basic Theory of Acoustic Emission

**Figure 1:**
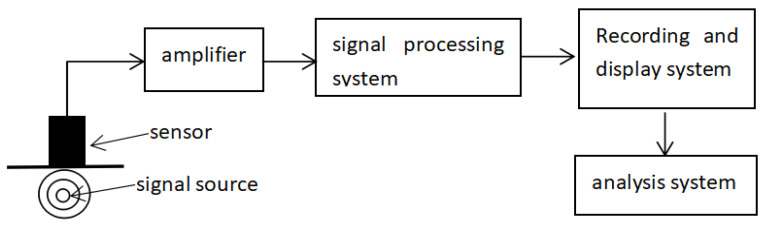
Acoustic emission acquisition principle.

When damage occurs in materials, a large amount of deformation energy is released and transient elastic waves are produced, which is called AE. An acoustic emission system collects these elastic waves by placing sensors on the surface of materials and converts them into electric signals, which are amplified by electric signal amplifiers. Finally, a computer receives these electric signals and records them. The principle of acoustic emission acquisition is shown in Figure 1.

**Figure 2:**
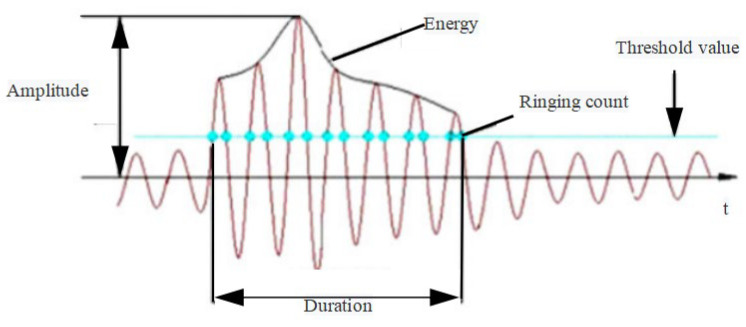
Acoustic emission damage waveform.

The acoustic emission signal waveform of a damage event is shown in Figure 2. The amplitude is the peak value of the waveform, which reflects the size of the damage. Each time the acoustic emission signal reaches the threshold value, it is a ringing count. The more ringing counts that occur each time the damage occurs, the more active the damage is. The acoustic emission energy is the area under the envelope of the waveform that exceeds the threshold value. This is not the fracture energy in the intuitive sense, but the larger the acoustic emission energy is, the larger the waveform area above the threshold value is, which indirectly reflects the degree of structural damage.

### 2.2. Basic Theory of Acoustic Emission

**Figure 3:**
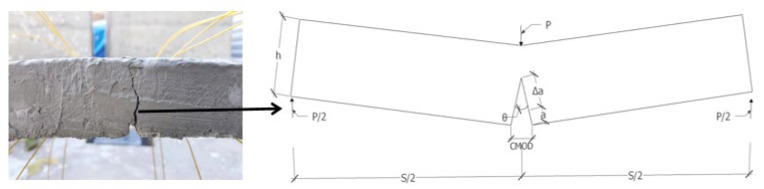
Schematic diagram of segment CMOD.

The segment crack opening displacement CMOD is shown in Figure 3. Assuming that the small deformation hypothesis holds, the segment structure rotates along the top point of the equivalent virtual crack. The segment CMOD expression is shown in Equation (1):

(1) $$\theta =\frac{CMOD}{\Delta a+a}$$

where *θ* is the crack rotation angle; *a* is the original crack length; Δ*a* is the newly generated crack length.

Regardless of energy loss other than damage, it is assumed that all work conducted by the load is used for segment cracking, the segment fracture energy is expressed in Equation (2):

(2) $$W=\int_{0}^{\theta_{0}}Md\theta$$

where *M* is the bending moment at the crack interface.

Since, *θ* is a function of CMOD, let *θ* = f(CMOD). Then Equation (2) can also be expressed as Equation (3):

(3) $$W=\int_{0}^{CMOD_{0}}M\mathrm{df}\left(\mathrm{CMOD}\right)$$

The fracture energy can directly reflect the bearing capacity and fracture damage degree of the segment, so it is reasonable and feasible to use CMOD to describe the degree of cracking of the segment.

## 3. Trial Overview

### 3.1. Specimen Design

In order to explore the evolution characteristics of segment cracks, this test relies on a subway shield tunnel. The outer and inner diameters of the segment are 6.2 m and 5.5 m respectively, the thickness of the segment is 0.35 m, and the width is 1.2 m. The concrete is made of C50 waterproof concrete with a waterproof grade of P12, and the main reinforcement are HRB335 steel bars with a reinforcement ratio of 0.9%. According to C_l_ = 1:12, bulk density similarity ratio C_γ_ = 1:1; friction angle, Poisson’s ratio, strain similarity ratio C_φ_ = C_μ_ = C_ε_ = 1; model strength, stress, cohesion, elastic modulus similarity ratio C_R_ = C_σ_ = C_c_ = C_E_ = 12, five groups of segment model specimens are configured. In order to be closer to the prototype material, the test model specimen was made of concrete. According to the Ordinary Concrete Mix Proportion Design Regulations (JGJ 55-2011) [22], concrete similar materials should be selected with a high water-cement ratio (1.3), high sand ratio (50%), low elastic modulus (the maximum particle size of gravel is 5 mm), low-grade cement (R325) and micro-concrete, configured according to the ratio of cement:river sand:crushed stone:water = 1:5.5:5.5:1.3. The physical parameters of the segment prototype and model are shown in Table 1.

### 3.2. Loading Method and Measurement Arrangement

**Figure 4:**
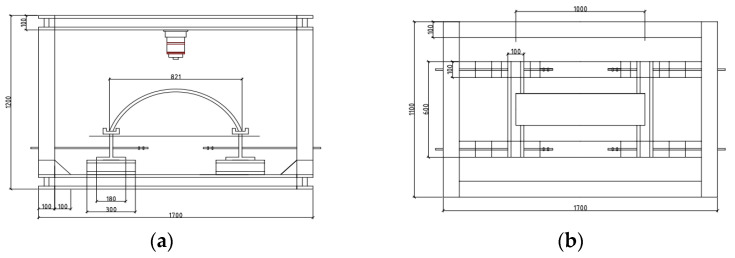
Model loading test device: (**a**) front view; (**b**) top view.

The self-designed “structural bending test system” was used as the loading device for the test, which could adjust the horizontal distance of the bottom support and was suitable for bending tests of specimens of different sizes. Its structural design is shown in Figure 4. According to The *Standard for Test Methods of Concrete Structures (GB/T 50152-2012)* [23], the test is performed by displacement-controlled loading, with 0.5 mm loading at each level until the component is destroyed.

**Figure 5:**
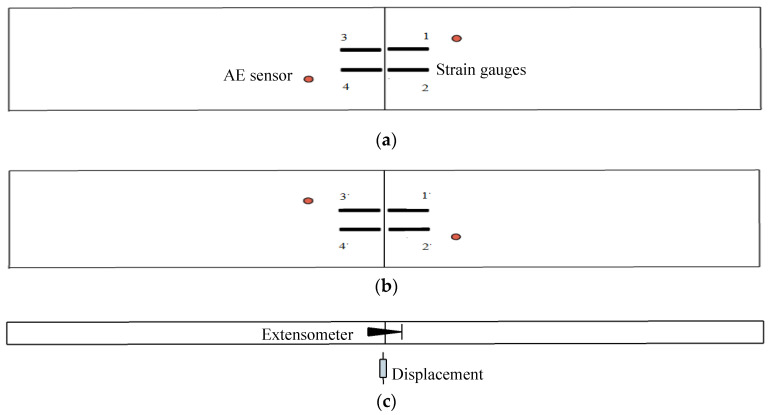
Sensor location: (**a**) Strain Gauge and Sensor Arrangement (outside); (**b**) Strain Gauge and Sensor Arrangement (inside); (**c**) Displacement and Extensometer.

In order to accurately identify segment crack damage evolution, acoustic emission time parameters, sound velocity, and threshold value, other detection parameters are set according to existing research results [15]. Considering the principle that acoustic emission sensors are wrapped around the specimen, four acoustic emission sensors are arranged around the specimen. At the same time, strain variation, displacement meter, and extensometer were used to record the internal force and response changes of the segment during the whole test process. The layout of each detection point is shown in Figure 5.

## 4. Test Results and Analysis

### 4.1. Crack Failure Process

**Figure 6:**
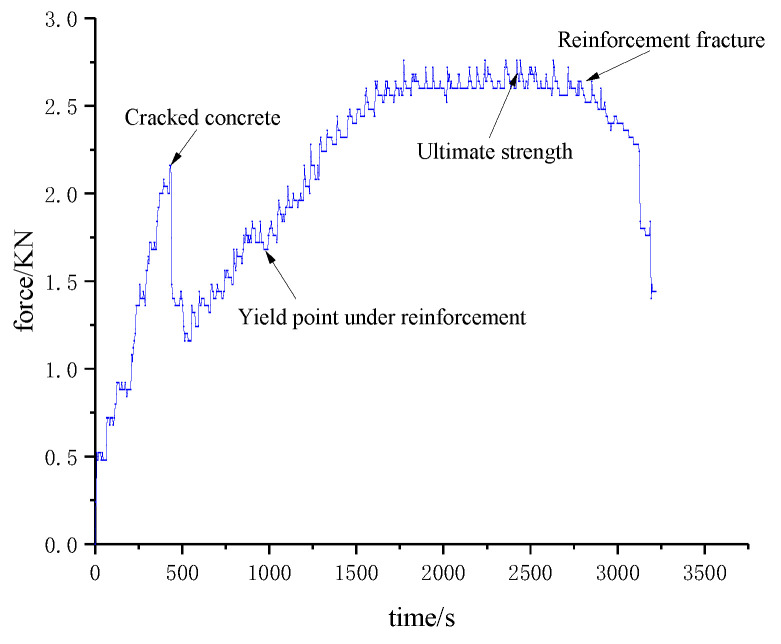
Force-time curves.

**Figure 7:**
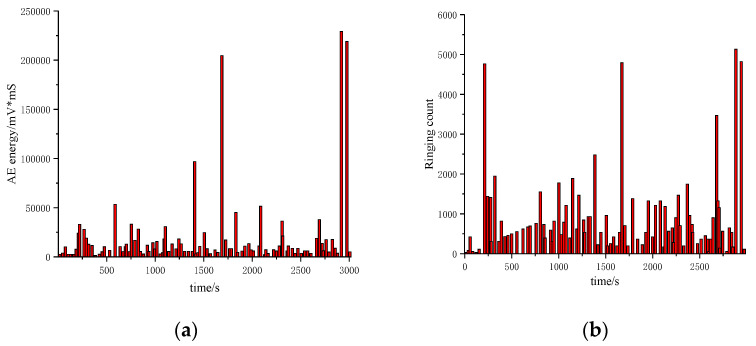
Acoustic emission acquisition results: (**a**) Acoustic emission energy; (**b**) Ringing count.

Segment crack damage actually starts from mesoscopic damage, and the occurrence of micro-damage is always accompanied by the generation of a sound emission signal. Therefore, a high-performance acoustic emission device was used to record all acoustic emission signals of the model test. Crack damage is a variable with time as the index. In order to describe the whole process of segment fracture and explore the characteristics of fracture in each period more accurately, this paper uses time as the benchmark to capture acoustic emission energy and ringing count by the transient method. The force-time curves of the segment and the acoustic emission detection results are shown in Figure 6 and Figure 7.

According to the variation trend of the results in the figure, the evolution of segment crack damage can be divided into three stages: concrete fracture stage in tension zone, reinforcement yield strengthening stage, and reinforcement fracture structure failure stage.

It can be seen from Figure 6 and Figure 7 that the acoustic emission energy and ringing count results have local sudden changes at some time nodes, and the timing of occurrence is basically the same as the time node of the sudden change of the segment force. When concrete cracks, the acoustic emission energy reaches 36.57 × 10^3^ mV*mS, and the ringing count increases to 47.46 × 10^2^. When the steel bar passes the yield point and enters the strengthening stage, the acoustic emission energy and ringing count reach 20 × 10^4^ mV*mS and 45.23 × 10^2^, respectively. After the steel bar fracture, the structure completely lost its bearing capacity. At this time, the acoustic emission energy and ringing count increased to 22.50 × 10^4^ mV*mS and 50.00 × 10^2^, respectively, reaching the peak value in the whole test process. It shows that the acquisition results of acoustic emission can directly reveal the mechanical state and damage information of the structure during the loading process.

### 4.2. Analysis of Microscopic Characteristics of Segment Crack Damage

**Figure 8:**
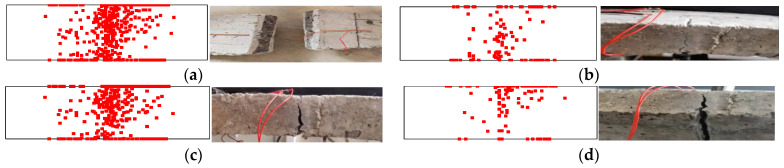
Acoustic emission identification results: (**a**) the final result of damage location; (**b**) concrete fracture stage in tension zone; (**c**) reinforcement yield strengthening stage; and (**d**) reinforcement fracture structure failure stage.

**Figure 9:**
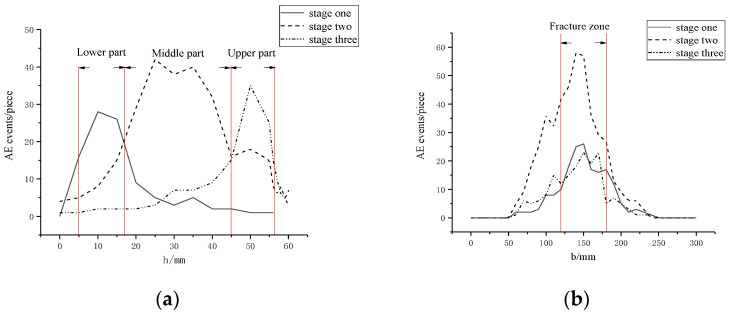
Location distribution of fracture damage: (**a**) longitudinal distribution; (**b**) transverse distribution.

The acoustic emission identification results are shown in Figure 8. The left side shows the acoustic emission localization results, and the right side shows the actual failure of the specimen. The damage location distribution is shown in Figure 9. It can be seen from the figure that there are significant differences in acoustic emission location results in the three periods of segment crack damage.

In the concrete fracture stage in the tension zone, the concrete in the lower part of the structure is the first to crack due to the tensile force on both sides. The acoustic emission location is more concentrated in the middle and lower parts, as the concrete in the lower part of the structure is first subjected to the tensile force to both sides, the concrete in the lower part cracks first, and the acoustic emission location is more concentrated in the middle and lower parts. Acoustic emission identification points are mainly concentrated in the range of 0–0.2 h (h is the thickness of the model, the same below) and 0.1 b (b is the width of the model, the same below) on each side of the crack.

In the reinforcement yield strengthening stage, the stress of the structure is mainly borne by the reinforcement, and the damage mostly occurs near the contact surface of the reinforcement and concrete. The cracks on the surface of the structure begin to develop upward, so the acoustic emission identification points are mainly concentrated in the range of 0.2–0.8 h and 0.1 b of each side of the crack. 

In the reinforcement fracture structure failure stage, the penetration crack is basically formed. The structure only relies on the upper steel bar and concrete to bear the force. The damage mostly occurs near the contact surface of the upper steel bar and concrete, so the acoustic emission identification points are mainly concentrated at 0.8–1.0 h and within 0.1 b of each side of the crack.

A total of 681 damage points were collected by the acoustic emission device during the whole test process, and the overall damage distribution was mainly concentrated near the actual cracks, which could effectively reflect the crack damage characteristics of each stage. It shows that the identification results of acoustic emission damage can effectively represent the damage location and damage development characteristics of structures.

In order to describe the characteristics of each stage of segment damage more clearly, the number of acoustic emission identification points, point exit rate, and unit volume density of each stage damage was calculated. The calculation results are shown in Table 2.

The results show that there are obvious numerical differences in each stage of segment crack damage. When the speed of identifying points is >1 s and the unit volume density of identifying points is >3.5 × 10^−4^/mm^3^, it can be determined that the structure is in the period when cracks start to open; maintenance and simple repair of the structure are required at this time. When the speed of identifying points is >0.2 s and the unit volume density of identifying points is >3.0 × 10^−4^/mm^3^, it can be determined that the steel bars inside the structure have tended to break, and at the same time, cracks have basically formed on the surface. At this time, the structure needs to be reinforced immediately when the speed of identifying points is >0.6 s and the unit volume density of identifying points is >3.5 × 10^−4^/mm^3^. It can be determined that the structure has basically tended to the edge of damage. The site should be withdrawn in time, and the structure should be demolished or rebuilt.

### 4.3. Analysis of Macroscopic Characteristics of Segment Cracks

Acoustic emission acquisition results characterize the internal microscopic level, while the generation of surface cracks is the macroscopic manifestation of the tube. In order to further characterize the whole process of segment cracking, this paper uses CMOD as the characterization index of macroscopic cracks.

**Figure 10:**
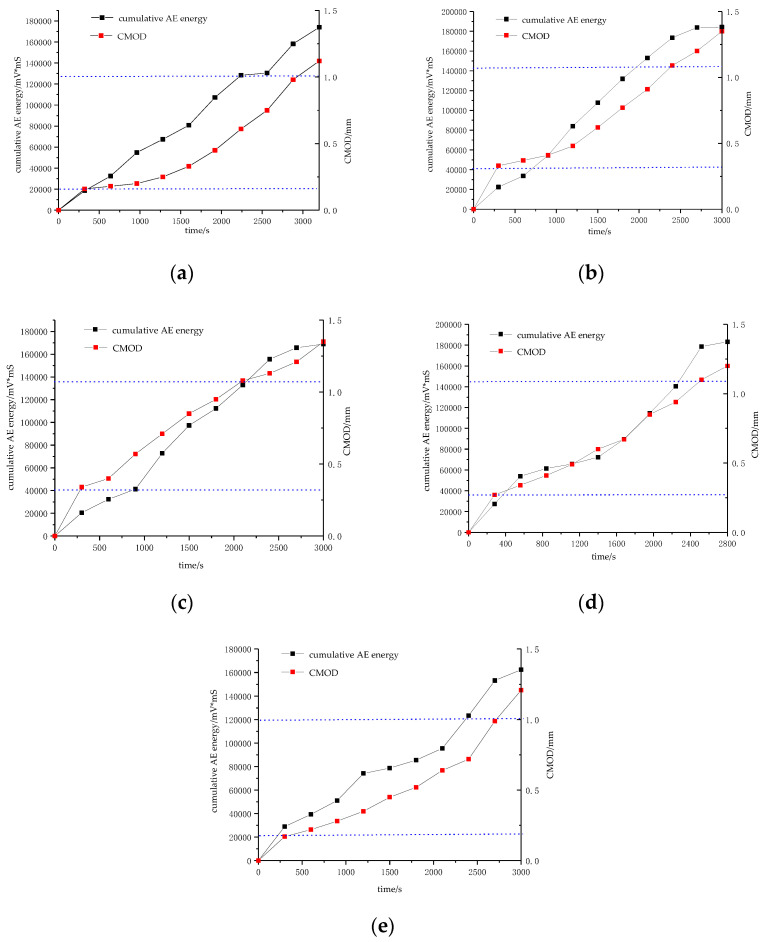
CMOD and cumulative energy change curve: (**a**) Test piece one; (**b**) Test piece two; (**c**) Test piece three; (**d**) Test piece four; (**e**) Test piece five.

The curve of CMOD variation of the segment and cumulative acoustic emission energy under load was plotted in Figure 10 through extensometer measurement. It can be seen from the figure that the change trends of the CMOD curve and the cumulative acoustic emission energy are basically the same, and they both increase approximately linearly. At the initial stage of loading, CMOD and cumulative acoustic emission energy curve will show a period of rapid growth, the crack width increases to about 0.25 mm, and the cumulative acoustic emission energy increases to about 22.00 × 10^3^ mV*mS, indicating that the concrete in the tension zone breaks at this time. After that, the slopes of the CMOD and cumulative acoustic emission energy curves decreased, the steel bars began to act as the main force at this time, and the restraint effect of the steel bars on the concrete increased. As the load increases, the slope of the CMOD and acoustic emission cumulative energy curves show an inflection point, CMOD is stable at about 1.1 mm, indicating that the steel bar has yielded at this time, and the restraint capacity of the steel bar on the concrete is reduced. Continuing to load, the slope of the CMOD curve and the acoustic emission cumulative energy curve increased rapidly at first, then tended to be flat, and finally reached the maximum value. It shows that at this time, the fracture of the steel bar completely loses the restraint effect on the concrete, the structure completely loses the ability to resist deformation, and a through crack has been formed on the surface of the segment.

## 5. Segment Cracking Damage Model

### 5.1. Damage Model Based on Acoustic Emission

The process of segment cracking is inevitably accompanied by the acoustic emission phenomenon. According to the analysis, the acoustic emission energy has a high correlation with the degree of cracking event. In this paper, the acoustic emission energy is used to evaluate the degree of segment cracking. Since the structure can recover by itself in the elastic failure stage and will not cause fatal damage to the structure, this paper focuses on the cracking characteristics of each stage after elastic failure.

Ohtsu et al. [24] combined the rate process theory and acoustic emission technology, and proposed a probability density function based on the stress level and the number of acoustic emission events:

(4) $$f\left(V\right)dV=dN/N_{0}$$

where, *V* is the relative load borne by materials, that is, the ratio of load borne by components and ultimate load; *N* is the number of acoustic emission events occurring at the current moment, that is, the number of acoustic emission ringing counts at the current moment; *N*_0_ is the number of acoustic emission events occurred when the component reaches peak stress, that is, the number of acoustic emission ringing at peak stress.

It has been analyzed above that the acoustic emission energy and the acoustic emission ringing count have the same variation law in the whole process of cracking. The energy information collected by the acoustic emission can characterize the degree of segment cracking. Therefore, in this paper, acoustic emission energy *G* at the current moment and *G*_0_ at the peak stress moment are used to replace *N* and *N*_0_ respectively. The corresponding probability density function can be obtained:

(5) $$f\left(V\right)dV=dG/G_{0}$$

The new probability density function can be obtained by dividing Equation (5) by *dV*:

(6) $$f\left(V\right)=\frac{1}{G_{0}}\frac{dG}{dV}$$

Integrating Equation (5) from the relative stress level 0 to 1:

(7) $$\int_{0}^{1}f\left(V\right)dV=\int_{0}^{G_{0}}dG/G_{0}=1$$

When the stress level is *V*, the cracking degree of the segment is expressed as:

(8) $$D=\int_{0}^{V}f\left(V\right)dV=\int_{0}^{V}\frac{1}{G_{0}}\frac{dG}{dV}$$

According to Formula (8), in order to establish the damage variable model with acoustic emission energy *G* as the variable, the functional relationship between acoustic emission energy and stress level should be found first. 

Weibull function is commonly used to describe the distribution of failure data of brittle materials. Therefore, the “S” shaped Weibull function was adopted in this paper to fit the changes of cumulative acoustic emission energy after the elastic stage of five groups of segment model specimens under load, the fitting function is expressed as:

(9) $$y=a-\left(a-b\right)\exp\left(-{\left(kx\right)}^{d}\right)$$

where, *a* is the maximum value of accumulated energy; *b* is the instantaneous energy value of segment cracking under load; *k* is the energy accumulation factor, the higher the value of *k*, the faster the accumulation of energy; and *d* is the comprehensive influence factor of energy.

**Figure 11:**
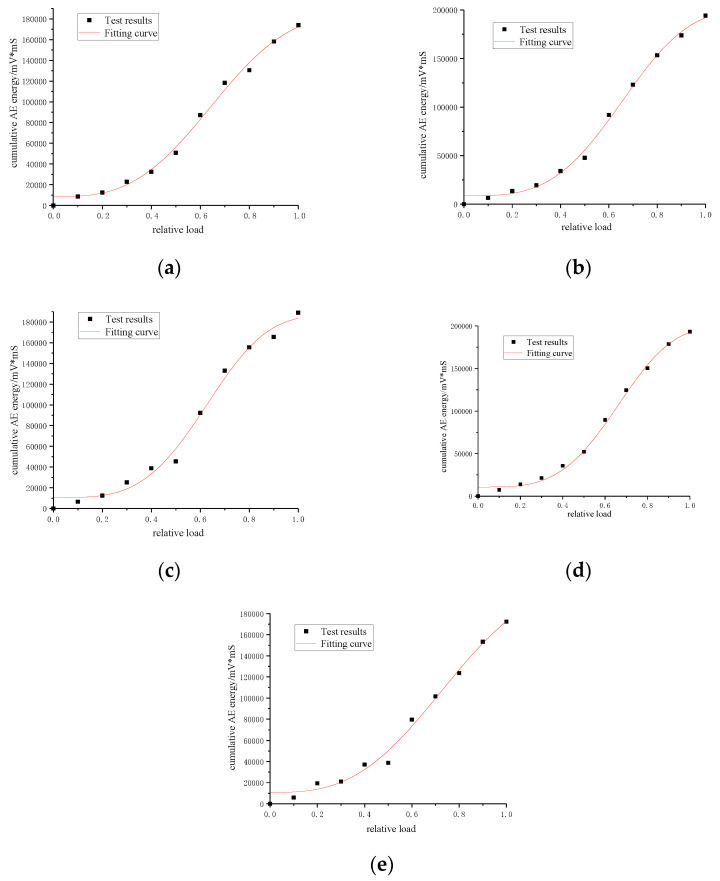
Cumulative energy-relative load fitting curve: (**a**) Test piece one; (**b**) Test piece two; (**c**) Test piece three; (**d**) Test piece four; (**e**) Test piece five.

The fitting curves of five groups of segmented specimens are shown in Figure 11, and the calculation results of each parameter are shown in Table 3. All R2 are greater than 0.95. It can be seen that this function can effectively describe the relationship between cumulative energy and relative load, which is:

(10) $$G=X\left(V\right)=a-\left(a-b\right)\exp\left(-{\left(kV\right)}^{d}\right)$$

Formula (10) is substituted into Formula (8) to obtain the expression of damage variable based on the accumulated acoustic emission energy:

(11) $$D=\left[a-\left(a-b\right)\exp\left(-{\left(kV\right)}^{d}\right)\right]/G_{0}$$

**Figure 12:**
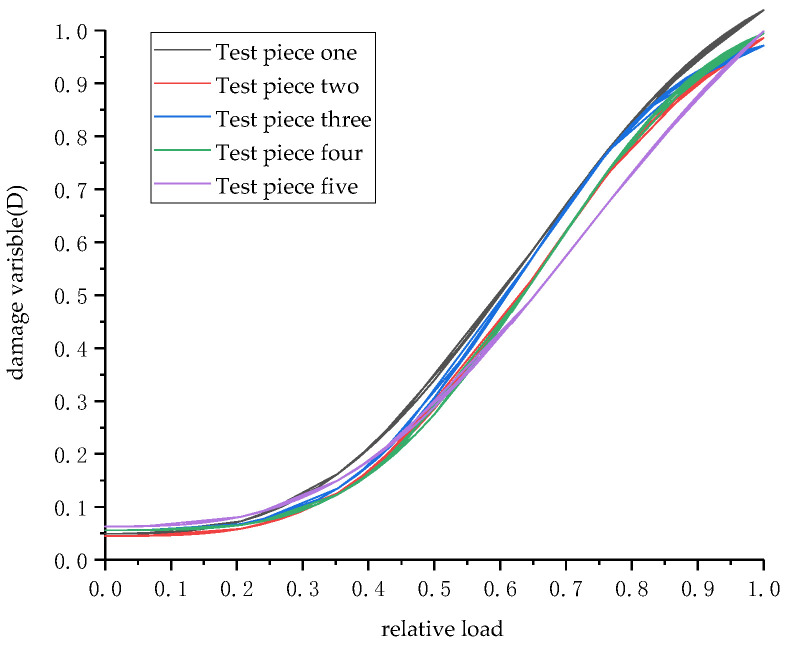
Damage evolution curve.

The crack loss evolution curves of each segment after elastic failure calculated by Formula (11) are shown in Figure 12. The crack loss evolution curves of five groups of segmented specimens are basically the same, indicating that the selection of the relation function between accumulated energy and relative load is reasonable. Since the crack evolution in the elastic failure stage is not considered in this paper, and Formula (10) shows that when *V* = 0, *D* = *b* ≠ 0, so the segment damage variable at the beginning of the curve should not be 0. In the initial stage of crack damage, the evolution speed of crack damage should not be large and the damage degree should not be high due to the restraining effect of steel bars on concrete. With the increase of the load, the reinforcement yields and the concrete is constrained to decline. At this time, the segment crack evolution speed should increase and the damage degree also further increases. Finally, the reinforced fracture structure completely loses its ability to resist deformation. At this time, the damage variable D approaches 1 and the damage degree reaches its maximum at the last stage of fracture evolution. The calculated fracture damage evolution curve can reasonably reflect all the characteristics of the actual segment crack damage evolution. It shows that the segment fracture damage evolution model based on accumulated energy of acoustic emission can effectively reflect the segment crack damage degree.

### 5.2. Correlation between CMOD and Damage Variables

**Figure 13:**
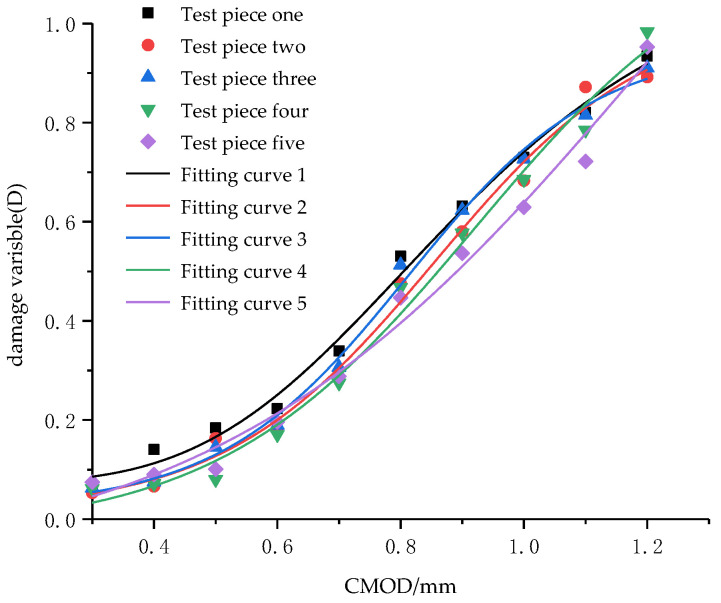
Relationship between CMOD and damage variables.

Crack is a macroscopic damage phenomenon developed from the segment crack. The more serious the segment crack is, the larger the surface crack propagates. As an important index of surface crack, CMOD can directly reflect the development of segment surface crack, but cannot quantitatively characterize segment crack damage degree. In order to quantitatively characterize segment crack damage degree by CMOD, the relationship between the CMOD value and the damage variable calculated by Equation (11) is plotted in Figure 13 and fitted by the function y = −(a − b)exp(−(kx)^d^). It can be seen from the figure that there is a good relationship between damage variables of five groups of segmented specimens and CMOD, and the five curves are basically the same. After concrete cracking, the reinforcement restrains the trend of concrete cracking, and the development of cracking loss is restrained. CMOD is stable at about 0.4 mm, and the damage variable is maintained at 0.15–0.2 at this time. The segment crack degree is not high, and only through reinforcement and repair, the segment can meet the requirements of continuous work. After the rebar yields, the restraint on the concrete decreases, and the cracking of the concrete intensifies. At this time, the surface crack develops to the through-crack, and the CMOD increases steadily and finally reaches about 1.1 mm, it has lost the ability to continue working. Continuing to load, the steel bars are broken, and through-cracks are formed on the surface; both CMOD and damage variable reached the maximum value. The results show that this function can be used to judge segment crack damage degree by CMOD, and then predict the overall structural stability of the segment.

## 6. Conclusions

The segment cracking changes in three stages, namely the concrete fracture stage in the tension zone, the reinforcement yield strengthening stage, and the reinforcement fracture structure failure stage. During the test, the acoustic emission signal suddenly changed a lot, and the damage increased rapidly. The acoustic emission ringing count and energy can effectively reflect the three-stage change characteristics of segment cracks, which provides a favorable basis for effectively analyzing the evolution characteristics of segment cracks.

(1) Acoustic emission identification results can effectively locate the location of structural damage, and through numerical analysis of data points, the numerical boundary of each stage of fracture damage is defined. The damage is mainly concentrated in the range of 0.1b on both sides of the crack. The yield strengthening stage is an important stage of fracture damage evolution, in which the damage data points account for 76.83% of all the damage data points. The data point exit rate is 0.225 s, and the data point density in the damaged area is 3.219 × 10^−4^/mm^3^.
(2) A crack damage evolution model, based on acoustic emission cumulative energy, was established. This can accurately calculate the damage variable of segment cracking failure and evaluate the evolution law of segment cracking damage.
(3) It is proved that the variation trend of CMOD is basically consistent with the variation trend of acoustic emission cumulative energy, and the quantitative relationship between CMOD and segment crack damage degree is established. It provides a feasible method to evaluate segment damage degree based on segment surface disease characteristics.

## Figures and Tables

**Table 1:** Physical parameters of segment model.

Classification	Standard Compressive Strength (fcu, k/MPa)	Elastic Modulus (E/GPa)	Rebar Yield Strength (δs/MPa)	Tensile Strength (σb/MPa)
archetype	50	34.5	335	300
model	4.6	2.63	34.12	23.9

**Table 2:** Comparison of acoustic emission detection results at different stages.

	Segment Fracture Stage		Number of Identification Points (PCS)	Identification Point Out Point Speed (units/s)	Identification Point Unit Volume Density (PCS/mm^3^)
Acoustic emission test results	Concrete fracture stage in tension zone	elastic stage	27	0.054	1.023 × 10^−4^
		Tensile concrete fracture	101	1.010	3.826 × 10^−4^
	Reinforcement yield strengthening stage	rebar yield	115	0.230	1.742 × 10^−4^
		Tensile limit of reinforcement	310	0.221	4.697 × 10^−4^
	Reinforcement fracture structure failure stage	Before the rebar breaks	36	0.052	1.364 × 10^−4^
		Structural instability due to reinforcement fracture	93	0.620	3.523 × 10^−4^

**Table 3:** Curve fitting results.

Specimen	a	b	d	k	R^2^
one	195,765.61	8583.83	3.03	1.36	0.99306
two	201,933.46	8776.07	3.41	1.37	0.99583
three	187,273.45	10,586.52	3.63	1.45	0.98603
four	200,377.23	8724.88	3.64	1.37	0.99261
five	209,565.19	10,855.97	2.92	1.19	0.98741

## Data Availability

The data presented in this study are available on request from the corresponding author.
